# CRI iAtlas: an interactive portal for immuno-oncology research

**DOI:** 10.12688/f1000research.25141.1

**Published:** 2020-08-24

**Authors:** James A. Eddy, Vésteinn Thorsson, Andrew E. Lamb, David L. Gibbs, Carolina Heimann, Jia Xin Yu, Verena Chung, Yooree Chae, Kristen Dang, Benjamin G. Vincent, Ilya Shmulevich, Justin Guinney

**Affiliations:** 1 Sage Bionetworks, Seattle, WA, 98101, USA; 2 Institute for Systems Biology, Seattle, WA, 98109, USA; 3 Anna-Maria Kellen Clinical Accelerator, Cancer Research Institute, New York, NY, 10006, USA; 4 University of North Carolina, Chapel Hill, NC, 27599, USA

**Keywords:** genomics, cancer, immunology, systems biology, R, Shiny

## Abstract

The Cancer Research Institute (CRI) iAtlas is an interactive web platform for data exploration and discovery in the context of tumors and their interactions with the immune microenvironment. iAtlas allows researchers to study immune response characterizations and patterns for individual tumor types, tumor subtypes, and immune subtypes. iAtlas supports computation and visualization of correlations and statistics among features related to the tumor microenvironment, cell composition, immune expression signatures, tumor mutation burden, cancer driver mutations, adaptive cell clonality, patient survival, expression of key immunomodulators, and tumor infiltrating lymphocyte (TIL) spatial maps. iAtlas was launched to accompany the release of the TCGA PanCancer Atlas and has since been expanded to include new capabilities such as (1) user-defined loading of sample cohorts, (2) a tool for classifying expression data into immune subtypes, and (3) integration of TIL mapping from digital pathology images. We expect that the CRI iAtlas will accelerate discovery and improve patient outcomes by providing researchers access to standardized immunogenomics data to better understand the tumor immune microenvironment and its impact on patient responses to immunotherapy.

## Introduction

Immuno-oncology (IO) is one of the most promising areas of cancer research, with IO-based treatments demonstrating high efficacy within certain cancer types and subsets of patients ^1– 4^. To broaden the utility of these therapies to more patients, fundamental research is required to improve our understanding of tumor-immune interactions—allowing the next-generation of therapeutics and treatment strategies to emerge ^4^. Advances in the IO field are impeded by the inaccessibility of IO study data and results and lack of data standardization, limiting the ability to easily compare results across studies. This has led to the underutilization of existing data, unnecessary study duplication, and failure to achieve rapid consensus in the field ^5^. With the vast increase in the number and scope of IO projects expected in the coming years combined with widespread adoption of genomics and other high dimensional technologies, these problems will be compounded going forward.

We developed the Cancer Research Institute (CRI) iAtlas portal ( https://www.cri-iatlas.org) to integrate IO research data, with the goal of providing an interactive, exploratory hub for the IO research community. In doing so, we hope to improve the accessibility and utility of critical resources generated from IO studies. iAtlas is a set of analytic modules—hosted on the web—for studying interactions between tumors and the immune microenvironment. These modules allow researchers to explore associations among a variety of immune characterizations as well as with genomic and clinical phenotypes.

The initial release of iAtlas (April 5, 2018) provided a rich resource to complement analysis results from The Cancer Genome Atlas (TCGA) Research Network on the TCGA data set comprising over 10,000 tumor samples and 33 tumor types ^6^ (“The Immune Landscape of Cancer”; here referred to as “Immune Landscape”). This study identified six immune subtypes that span cancer tissue types and molecular subtypes, and found that these subtypes differ by somatic aberrations, microenvironment, and survival. Per-sample characterizations included total lymphocytic infiltrate (from DNA methylation as well as H&E imaging data), estimated cell type fractions, immune gene signature expression, MHC/HLA type and expression, antigen presentation machinery, T cell and B cell receptor repertoire inference, viral/microbial characterization, associations with pathway disruption and activity, and other analysis results. The Immune Landscape ^6^ manuscript reported on the most novel and potentially therapeutically salient statistical associations between these immune subtypes and the results of the immune characterization. We have continued to develop and evolve the CRI iAtlas application; here, we report the technical design and implementation of iAtlas up to and including the recently released version 1.2 ^7^. This version includes new features requested by users including: (1) user-defined loading of sample cohorts, (2) a tool for classifying expression data into immune subtypes, and (3) integration of TIL mapping from digital pathology images.

## Methods

### Implementation

iAtlas is a web-based application to enable data exploration for clinicians, biologists, and informaticists. The inputs and architecture of the application are described below.

### Data

The iAtlas app uses structured data and outputs from the Immune Landscape ^6^ study and the TCGA PanCancer Atlas initiative ^8^, which harmonized TCGA data, ensuring uniform quality control and sample inclusion, batch effect detection, normalization across platforms, combination mutation calling from multiple centers, and robustly compiled clinical and outcome data. A key source of iAtlas data is the table summarizing tumor-sample and immune characterizations for 11,080 TCGA patient participants of the TCGA, Table S1 of the Immune Landscape ^6^ manuscript, here termed the “PanImmune Feature Matrix”. Auxiliary data were sourced from files available on this manuscript’s data page at the NCI Genomic Data Commons, from the TCGA PanCancer Atlas Data Mirror, and from the TCGA PanCancer Atlas working space in Synapse (see *Data availability*). iAtlas data were formatted as data frames (tables) and stored as “ *feather*” files ( https://github.com/wesm/feather) on the application server for fast loading ( Table 1).

**Table 1.** iAtlas data files.

Filename	Description
fmx_df.feather	All immune readout features/variables (11,080) across samples (139).
feature_df.feather	Additional annotations for a subset (104) features.
feature_method_df.feather	Annotation of methods (21) used to compute features.
im_direct_relationships.feather	Annotation and source of immunomodulators genes (79).
im_expr_df.feather	Gene expression for immunomodulators genes (76) across samples (9,693).
im_potential_factors.feather	Additional genes (97) that have potential to be involved in immune modulation.
io_target_annotations.feather	Annotation of immuno-oncology target genes (405).
io_target_expr_df.feather	Gene expression for immuno-oncology target genes (401) across samples (9,693)
sample_group_df.feather	Annotation of the sample groups (137 - TCGA studies, TCGA cancer subtypes and Immune Subtypes).

**Annotation and browsing of the PanImmune Feature Matrix:** iAtlas includes a **Data Description** page with details on all variables presented in individual modules, with the ability for users to “drill down” on related groups of variables to understand how values were derived. Variables are listed in a text-searchable table containing the name of the variable, the ‘Variable Class’, the unit (if applicable), and whether the variable is numeric or categorical. A ‘Variable Class’ is the name of a group of variables that are of similar type and are often the result of one particular analysis. Clicking on a row exposes a list of all variables in the ‘Variable Class’ and provides links to text descriptions of the analysis methods used to generate the variables.

### R/Shiny architecture

**Figure 1.**
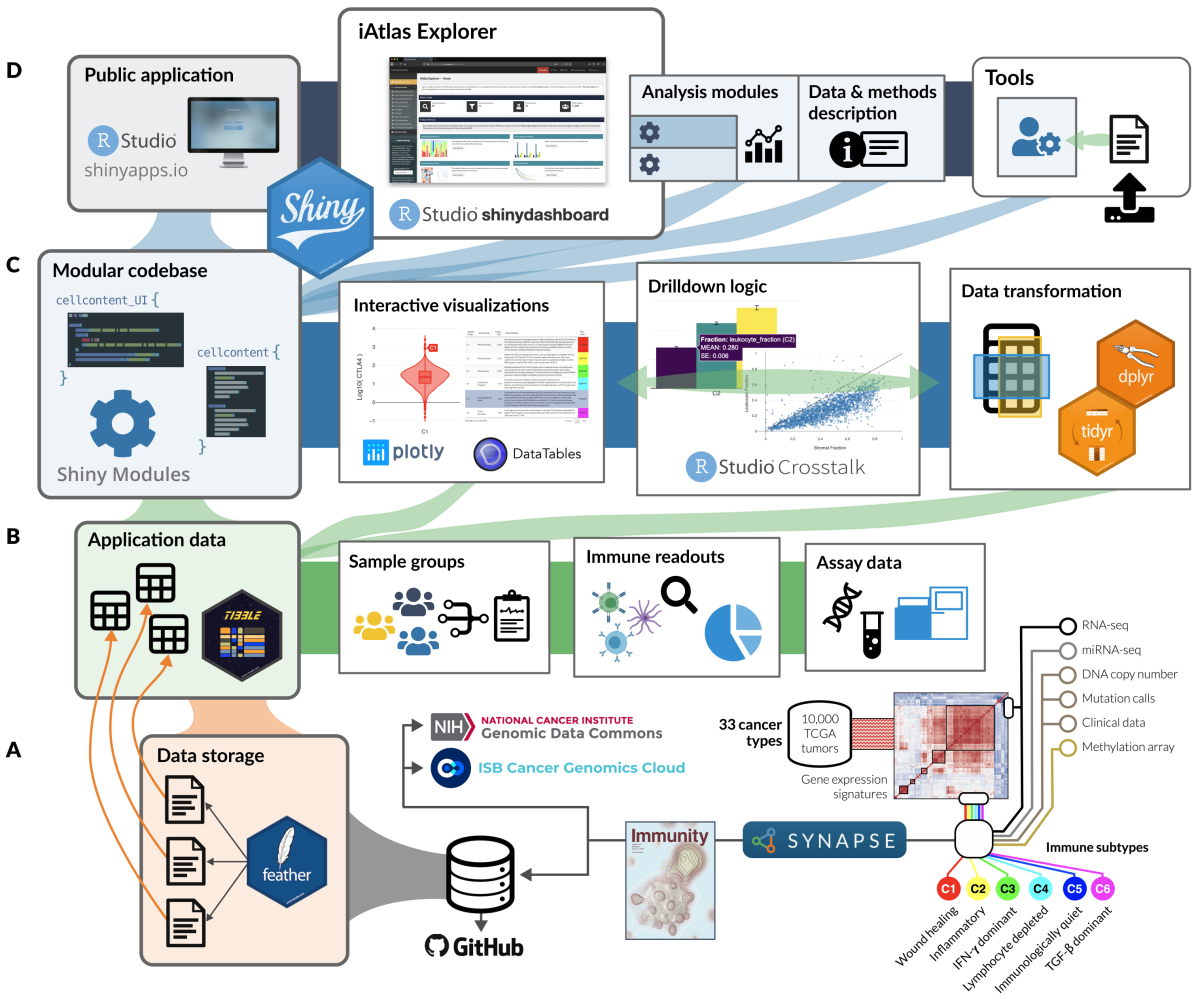
iAtlas architecture overview. ( **A**) Structured data from immunogenomic analyses, including the Immune Landscape ^6^ study and expanding over time, are organized and stored as flat (i.e., *feather*) files within Synapse and made available alongside the application code in GitHub. ( **B**) Tabular data from feather files are read from disk into memory to drive all operations related to sample groupings, sample-level immune characterizations (readouts), and more granular—and high dimensional—assay measurements. ( **C**) The core application code is built as a catalog of Shiny Modules, each of which encapsulates logic for data transformation and visualization related to a scientific theme or assay type. ( **D**) Analysis modules, tools, and data description views are hosted in a unified application on *shinyapps.io*; the layout and connectivity between modules in the **iAtlas Explorer** space are managed by the *shinydashboard* ^21^ library.

iAtlas is powered by Shiny ^9^ and makes extensive use of Shiny Modules ^10^ to organize code into composable units ( Figure 1). Each iAtlas **Analysis module** is designed as a Shiny module, allowing simple integration of new analytical functionality. iAtlas uses the tidyverse ^11^ family of R packages (e.g., dplyr ^12^, tidyr ^13^, purrr ^14^, stringr ^15^, tibble ^16^) as well as the wrapr ^17^ package to assist with tidy evaluation. These functions power the data transformations of internal tabular data that are then used to create the interactive plots (i.e., with the plotly ^18^ graphing library) and data tables (via the *DT* ^19^ wrapper to the *DataTables* library) seen through the iAtlas modules. We also make heavy use of the crosstalk ^20^ package to enable event-driven updates to the application state. The core iAtlas application is hosted on https://shinyapps.io.

### Analysis modules

**Figure 2.**
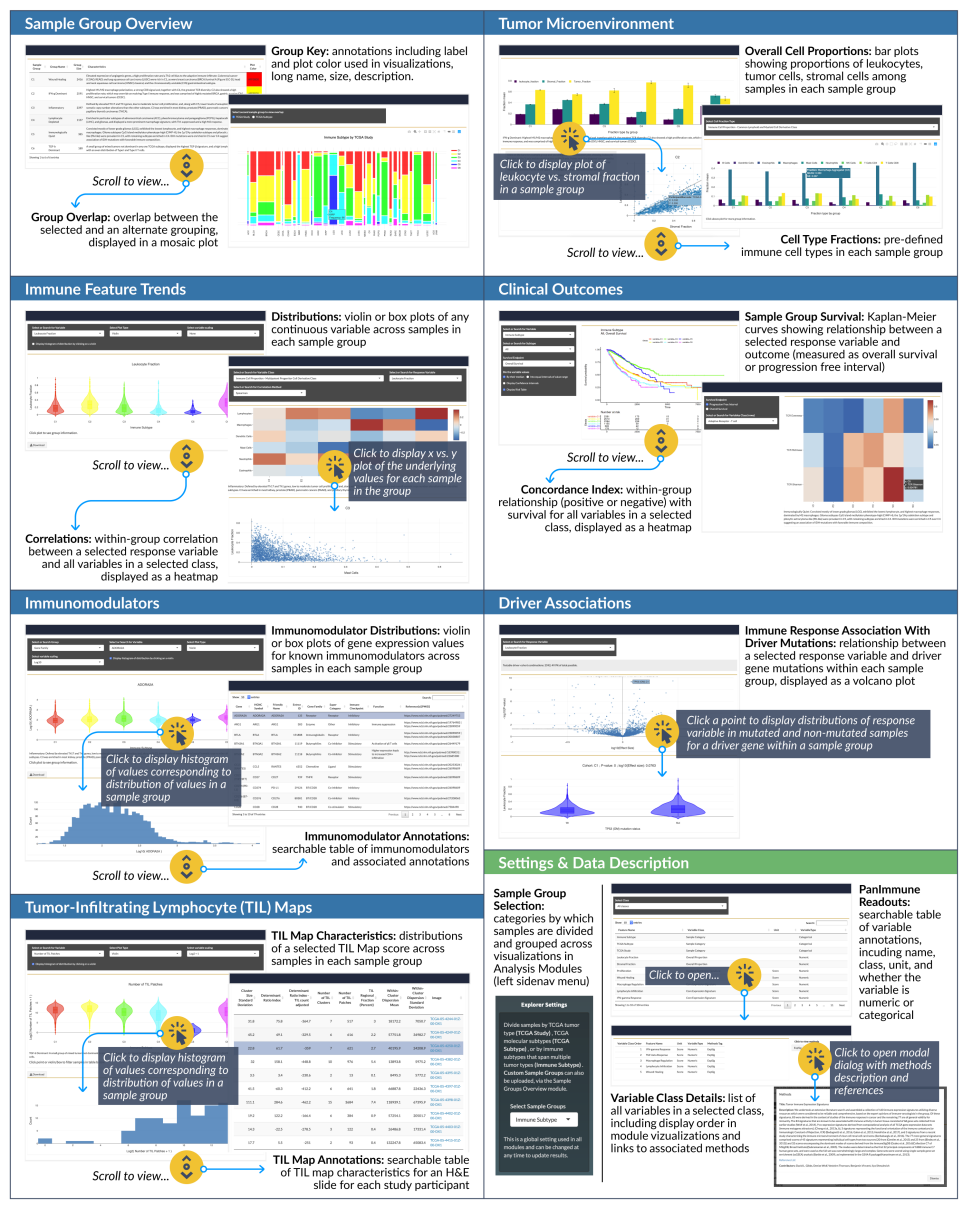
iAtlas Explorer. A range of **Analysis modules** (blue boxes above) are available that span from clinical to molecular and imaging data types. Within each module, interactive controls allow researchers to expand views, exposing underlying data and results. Settings are available (green box above) to select the sample groupings ( TCGA Study, Disease Subtype, or Immune Subtype) which then propagate through modules.

The main feature of the iAtlas interface is the **iAtlas Explorer** ( Figure 2, found under the **EXPLORE** tab), which provides several Analysis modules to explore and visualize results. Each module supports a type of exploration, with interactive views and controls to enhance and extend the results and analytics as initially described in the Immune Landscape ^6^ study. The layout of pages and sections within the iAtlas Explorer is driven by the shinydashboard ^21^ package.

Within each module in iAtlas, results are displayed relative to Sample Groups, corresponding to defined study cohorts. Several Sample Groups options are pre-loaded in the tool: first, TCGA tumor type (TCGA Study), which are the standard TCGA tumor types collected and designated by the TCGA. Second, TCGA tumor subtypes (TCGA Subtype), a compendium of further subdivision of TCGA studies into molecular subtypes according to publications by the TCGA Research Network ^22^. Finally, a division of tumor samples into distinct patterns of immune response in cancer (Immune Subtypes) is provided ^6^. The choice of Sample Groups is global across all modules but can be updated at any time via the **Select Sample Groups** element in the side menu. We also allow users to upload custom-grouped samples and analyze those with iAtlas modules. The selection of a sample group defines the samples utilized in all analysis modules. For convenience, group annotations can be displayed in visualizations within each module.

**Sample Group Overview:** View summary information for user-selected sample cohort groups. There are currently three sections: ****Custom Groups****, ****Group Key****, and ****Group Overlap****. Respectively, these sections permit loading of user-defined sample groups, review of detailed annotations of sample groups in a table, and display of overlap between different types of groupings in a mosaic plot.
**Tumor Microenvironment:** Explore immune cell proportions in sample groups with two sets of faceted bar charts, one for overall cellular proportions (i.e., leukocyte, stromal, and tumor fraction) and one for computed immune cell proportions (e.g., monocytes, CD8+ T cells, naive B cells).
**Immune Feature Trends:** Visualize how immune readouts vary across sample groups. Violin or box plots show the distribution of individual values across samples in each group, while heatmaps and scatter plots can be used to explore the correlation between any pair of variables within each group.
**Clinical Outcomes:** Quantify the relationship between immune response and disease outcome, in terms of either overall survival (OS) or progression free interval (PFI) ^23^. Results are displayed as Kaplan Meier plots as well as heat maps showing the concordance index between variables and survival.
**Immunomodulators:** Explore the expression of genes coding for immunomodulating proteins ^6^, which include therapeutically important immune checkpoint proteins. Immunomodulators are organized by grouping into three categories: Gene Family (such as *“TNF”*, *“MHC Class II”*, *“Immunoglobulin”*, or *“CXC chemokine”*), Super Category (such as *“Ligand”*, *“Receptor”*, or *“Antigen presentation”*), and Immune Checkpoint (classified as *“Inhibitory”* or *“Stimulatory”*). Violin and box plots are again used to present distributions, and a table provides additional metadata about immunomodulator genes.
**Driver Associations:** Test and visualize associations between mutations and IO-related response variables. In the Immune Landscape ^6^ study, we reported somatic driver alterations that are correlated with increases or decreases in overall immune cell content, or with the fraction of individual immune cell types. These and other variables can be selected to calculate the significance of relationships in each sample group and view results in a volcano plot.
**TIL Maps:** We used the results of a recently reported method to assess which spatial regions of hematoxylin and eosin (H&E) whole slide images show evidence of tumor-infiltrating lymphocytes (TILs) ^24^. The method, which uses deep learning, was applied to thousands of H&E slides of the TCGA, allowing slides to be characterized in terms of TIL density and patterns.

**Integration with Landscape of IO Drug Target Development:** CRI has compiled and published comprehensive overviews describing ongoing immunotherapy drug trials, including targets, agents, and tumor sites and has made summaries available in an online resource, the Immune-Oncology Landscape (IO Landscape ) ( www.cancerresearch.org/IO-landscape) ^25– 28^. The iAtlas and the IO Landscape resource have been interlinked, enabling researchers to more readily understand the relationship between targeted proteins in IO therapy and the behavior of those targets in tumor tissue.

In ***IO Target Gene Expression Distributions***, the distribution of gene expression values for the selected IO target, by sample group, is displayed in violin plots. Clicking on the expression distribution (violin plot) of a particular sample group, a histogram of the values is displayed.
The ****IO Target Annotations**** section provides a searchable table with IO targets and associated annotations. In the rightmost column, a link is provided to a view of the IO Landscape page, the selected target is highlighted in summary barcharts showing the number of agents and cancer types being studied for that target.

In the opposite direction, clicking on targets in the barcharts in the IO Landscape on CRI web pages brings up the target gene expression in iAtlas.

### Tools

**iAtlas Tools** are accessible via the **TOOLS** tab on the iAtlas Portal. Modules in this space of the portal enable users to “bring their own data” for processing through immunogenomic algorithms that drive some of the results presented in the Analysis modules described above.

**Immune Subtype Prediction:** This tool performs classification of RNA-seq data into one of six immune subtypes as described in the Immune Landscape ^6^ study. Using a new ensemble model based on XGBoost ^29^, researchers can upload their own data for classification ^30^. Each member of the ensemble was trained on a random subset of previously reported immune subtypes ^6^ and features (described below) based on gene expression data from the TCGA PanCancer Atlas Initiative ^8^. All code and methods have been confirmed as reproducible. An R package is available on GitHub ( https://github.com/CRI-iAtlas/ImmuneSubtypeClassifier) ^30^.

The submitted expression data—subsetted to the 485 genes that comprised the 5 signatures that produced the immune subtypes—are used to generate robust features of three types: quartiles, binary gene-pairs, and signature-pairs. For example, given a single sample, genes are binned into quartiles and given a bin label (quartile features). Then, similar to the “Top Scoring Pairs” classifier ^31^, genes are paired, and given binary values depending on whether ( *g _i_ > g _j_*) for two gene expression values, *g _i_* and *g _j_*. Lastly, signature-pair features are calculated using the five immune subtype signatures, where *s _mn_* = ∑ _*ij*_( *g _im_ > g _jn_*)/ *k*, where *g _im_* is gene *i* from signature *m*, *g _jn_* is gene *j* from signature *n*, and *k* is the number of gene pairs considered resulting in a value between 0 and 1. The features are computed independently for each sample, and do not require normalization across samples. These features are given to a trained XGBoost classifier which returns a probability of being in any of the six subtypes. Lastly, a “best call” is made with a final trained XGBoost classifier using the six probabilities as input. To validate the robustness of the classifier, TCGA data were processed using four different software pipelines and normalization, showing that classification performance was independent of the gene expression quantification method ^30^. Along with a downloadable table of results, visualizations are also provided. This tool is a convenient way for researchers to apply the methods of the Immune Landscape ^6^ study to their own data without difficult statistical coding.

## Operation

To use iAtlas, access the web app via https://www.cri-iatlas.org. The software can also be run locally on all platforms (Windows, Mac, Linux). To run the Shiny app locally, a working R installation with necessary libraries is required and an installation of RStudio is recommended.

To install and run the app locally:

1. Clone shiny-iatlas GitHub repository
(git clone> 
                                    https://github.com/CRI-iAtlas/shiny-iatlas)
2. Open shiny-iatlas.Rproj in RStudio
3. Install packages. In the RStudio console, run:
renv::restore()
4. Start the app by running:
shiny::runApp()

## Use cases

### Reproducing published results and gaining information on underlying data

**Figure 3.**
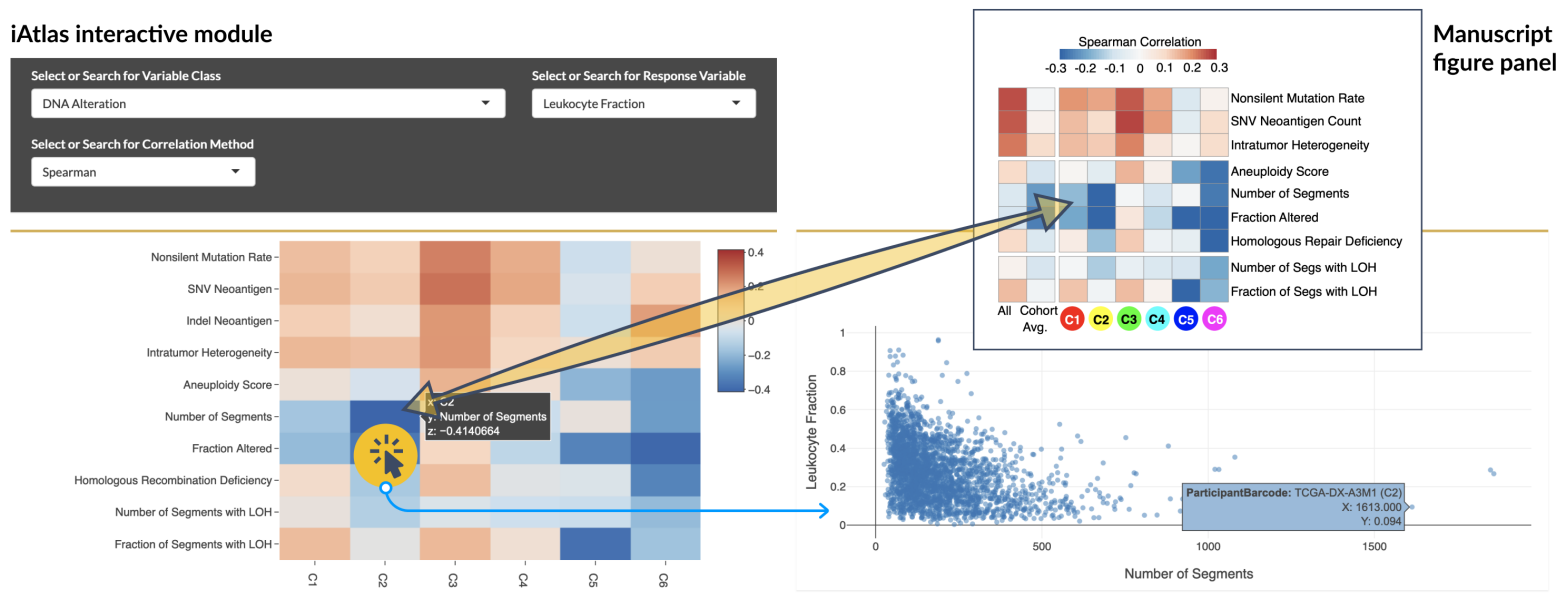
Visualization of the correlation of DNA damage in cancer with the degree of immune cell infiltration. *Top right:* Original manuscript figure panel from the Immune Landscape ^6^study. *Bottom left:* Equivalent figure generated in iAtlas. By selecting a specific heatmap cell (highlighted), the underlying data is displayed ( *Bottom right*), using the selections shown. Individual points can be selected to get sample IDs and additional information (blue box).

**Figure 4.**
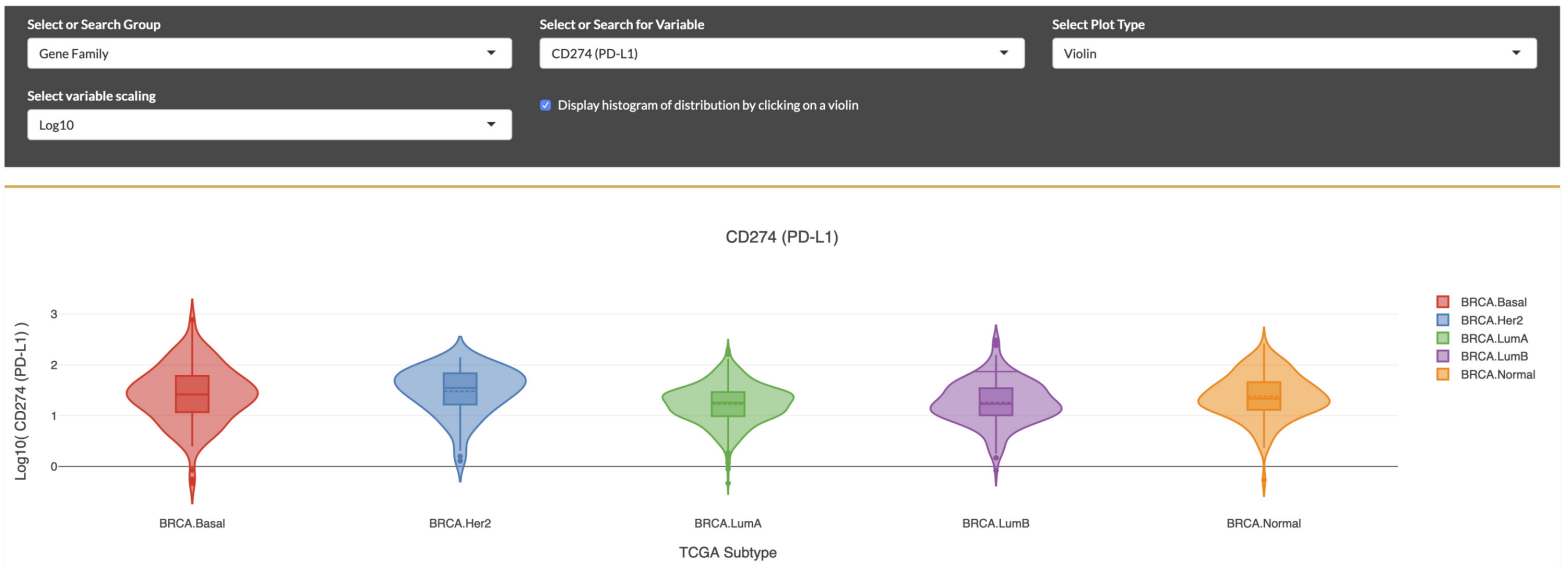
Visualization of the PD-L1 immunomodulator expression patterns in breast cancer subtypes. Selection for PD-L1 expression distributions displayed as violin plots within molecular subtypes of breast cancer, according to “PAM-50” classification. Elevated expression is seen in the HER2 subtype.

One of the initial motivations behind iAtlas was to provide an interactive platform that is able to reproduce figures published in the Immune Landscape ^6^ manuscript but expands that with the ability to generate variations of those figures, for other choices of tumor samples and immune readouts of interest. As an example, in order to reproduce Figure 4A from the Immune Landscape ^6^ publication, which shows the correlation of DNA damage measures with the fraction of leukocytes in the tumor, we began by selecting the **EXPLORE** tab. We then opened the **Immune Feature Trends** module and selected the *“Immune Subtype”* option under **Select Sample Groups** in the ****Explorer Settings**** panel ** in the left menu. In the ensuing module page, at the ****Correlations**** section ( Figure 3), we selected the *“DNA Alterations”* under **Select or Search for Variable Class**, *“Leukocyte Fraction”* under **Select or Search for Response Variable**, and the *“Spearman”* method under **Select or Search for Correlation Method** (each a separate dropdown menu). This produced a heatmap identical in content to Figure 4A in the Immune Landscape ^6^ publication. However, the heatmap provides additional information on underlying data via interactivity: by clicking on a heatmap-cell, the underlying data is displayed in a scatterplot. Hovering a cursor over a point in the scatter plot reveals sample-level information.

Table 2 lists the particular manuscript figures (from the Immune Landscape ^6^ publication) that can be reproduced or adapted to specific research questions.

**Table 2.** Immune Landscape figures in iAtlas. iAtlas Analysis module (Column 1) and examples of figure panels (Column 2) in the Immune Landscape ^6^ manuscript that can be generated using the module. Researchers can use this as a starting point for tailoring figures to their own interests.

iAtlas Analysis module	Immune Landscape figures
Sample Group Overview	1D, S1B
Tumor Microenvironment	2A, 2C, S2A
Immune Feature Trends	1C, 2B, 4A
Clinical Outcomes	3A, 3B, 3C, S3A, S3B, S3C
Immunomodulators	6B
Driver Associations	4D
TIL Maps	2D

### Exploring new IO results

With the iAtlas portal, scientists can explore and answer new questions based on specific research interests. For example, we asked: “ *What is the expression level of PD-L1, a therapeutically important protein, in subtypes of breast cancer?”* To answer this question, from the landing page, we first selected the *“TCGA Subtype”* sample group, followed by the *“Breast Invasive Carcinoma (BRCA)”* study subset. Next, we selected the **Immunomodulators** module ( Figure 4). Based on a very quick scan of the drop down, we didn’t see any names that matched our gene of interest, so we scrolled further down on the page to view the table of ‘Immunomodulator Annotations’. By typing in the first few letters of a gene name (e.g., “PD...”) into the ‘Search’ field, the table was filtered to a set of matching genes, and we could see that *“PD-L1”* is the Friendly Name for the gene *“CD274”* (the approved gene symbol on genenames.org). After returning to the **Select or Search for Variable** drop down menu above and selecting *“CD274 (PD-L1)”*, we were able to see a display of violin plots showing the distributions of gene expression across BRCA molecular subtypes. We could then visually compare distributions between subtypes, noticing for example the elevated expression level in the Her2 subtype compared to Basal breast cancer. These comparisons can guide further characterization not only of how gene expression can differ between TCGA subtypes of breast cancer, but also how these subtype-specific differences might correlate with clinical outcomes, as investigated in other studies ^32– 34^. Using this module and others, the researcher has the ability to answer new questions which could lead to developments in oncology research.

### Classification of immune subtypes on new data

In order to classify any tumor-derived gene expression samples into immune subtypes ^6, 30^, users can select the **TOOLS** tab (top right), which leads to an interface containing notes, several links and the controls. In order to classify new data, we submitted data as a text file, in this case tab separated, with the first column containing gene IDs and later columns containing samples. A provided example file can be found in the description text. The first row of the data was a header containing sample IDs. Gene IDs can be either HGNC gene symbols (preferred), Entrez ID, or Ensembl identifiers. The locally available data was selected using the *Browse* button, and the file delimiter was selected, along with gene ID type, using drop down menus. Hitting the *GO* button produced classifications, signature scores, and cluster probabilities, which were reported in a table that was downloaded as a csv, xlsx, or pdf file. In addition, a barplot with the frequency of predicted subtypes for the submitted data was displayed.

All data required to run the application and describe the Use Cases are available in GitHub and archived with Zenodo ^7^.

## Conclusions

CRI iAtlas is a platform that facilitates analysis and exploration of the tumor immune microenvironment by making IO-related data and tools accessible to the research community. iAtlas builds upon the comprehensive TCGA analysis of tumor-immune interactions on 10,000 tumors and illustrates how commonalities and differences of the immune response across 33 tumor types can provide clues for advancing therapeutics. iAtlas provides researchers with the tools to dive deeper into immunogenomic and clinical data and to develop and refine hypotheses regarding tumor-immune interactions that will empower researchers to gain insight and design the next generation of immuno-oncology treatment strategies.

## Data availability

### Source data

Original data files from the TCGA PanCancer Atlas publication can be found in the NCI Genomic Data Commons ( https://gdc.cancer.gov/about-data/publications/panimmune) or the TCGA PanCancer Atlas Data Mirror ( https://isb-cancer-genomics-cloud.readthedocs.io/en/latest/sections/PanCancer-Atlas-Mirror.html.

### Extended data

Zenodo: CRI iAtlas (Version 1.2.0). https://doi.org/10.5281/zenodo.3926757 ^7^.

Folder ‘Data’ contains all data required to run the application and describe Use Cases. This is also available on GitHub.

License: Apache License 2.0.

## Software availability

Source code is available from GitHub: https://github.com/CRI-iAtlas/shiny-iatlas.

Source code for the specific version described at the time of publication: https://github.com/CRI-iAtlas/shiny-iatlas/releases/tag/v1.2.0.

Archived source code at the time of publication: https://doi.org/10.5281/zenodo.3926757 ^7^.

Hosted iAtlas application on shinyapps.io: https://isb-cgc.shinyapps.io/shiny-iatlas.

Pinned version of the hosted iAtlas app described at the time of publication: https://isb-cgc.shinyapps.io/iatlas_v1-2.

License: Apache License 2.0.

## References

[1] Mellman I Coukos G Dranoff G: Cancer immunotherapy comes of age. *Nature.* 2011;480(7378):480–489. 10.1038/nature10673 22193102 PMC3967235

[2] Fridman WH Pagès F Sautès-Fridman C: The immune contexture in human tumours: impact on clinical outcome. *Nat Rev Cancer.* 2012;12(4):298–306. 10.1038/nrc3245 22419253

[3] Liu XS Mardis ER: Applications of Immunogenomics to Cancer. *Cell.* 2017;168(4):600–612. 10.1016/j.cell.2017.01.014 28187283 PMC5972371

[4] Binnewies M Roberts EW Kersten K: Understanding the tumor immune microenvironment (TIME) for effective therapy. *Nat Med.* 2018;24(5):541–550. 10.1038/s41591-018-0014-x 29686425 PMC5998822

[5] Baruch EN Berg AL Besser MJ: Adoptive T cell therapy: An overview of obstacles and opportunities. *Cancer.* 2017;123(S11):2154–2162. 10.1002/cncr.30491 28543698

[6] Thorsson V Gibbs DL Brown SD: The Immune Landscape of Cancer. *Immunity.* 2018;48(4):812–830.e14. 10.1016/j.immuni.2018.03.023 29628290 PMC5982584

[7] Eddy J Gibbs D Lamb A: CRI iAtlas (Version 1.2.0). *Zenodo.* 2020 10.5281/zenodo.3926758

[8] Hutter C Zenklusen JC: The Cancer Genome Atlas: Creating Lasting Value beyond Its Data. *Cell.* 2018;173(2):283–285. 10.1016/j.cell.2018.03.042 29625045

[9] Chang W Cheng J Allaire JJ: shiny: Web Application Framework for R. *R package version 140.* 2019 Reference Source

[10] Cheng W: Modularizing Shiny app code.2020 Reference Source

[11] Wickham H Averick M Bryan J: Welcome to the Tidyverse. *JOSS.* 2019;4(43):1686 10.21105/joss.01686

[12] Wickham H François R Henry L: dplyr: A Grammar of Data Manipulation. *R package version 083.* 2019 Reference Source

[13] Wickham H Henry L: tidyr: Tidy Messy Data. *R package version 100.* 2019 Reference Source

[14] Henry L Wickham H: purrr: Functional Programming Tools. *R package version 033.* 2019 Reference Source

[15] Wickham H: stringr: Simple, Consistent Wrappers for Common String Operations. *R package version 140.* 2019 Reference Source

[16] Müller K Wickham H: tibble: Simple Data Frames. *R package version 213.* 2019 Reference Source

[17] Mount J Zumel N: wrapr: Wrap R Tools for Debugging and Parametric Programming. *R package version 192.* 2019 Reference Source

[18] Sievert C Parmer C Hocking T: plotly: Create Interactive Web Graphics via “plotly.js”. *R package version 490.* 2019 Reference Source

[19] Xie Y Cheng J Tan X: X: DT: A Wrapper of the JavaScript Library “DataTables”. *R package version 09.* 2019 Reference Source

[20] Cheng J: crosstalk: Inter-Widget Interactivity for HTML Widgets. *R package version 100.* 2016 Reference Source

[21] Chang W Ribeiro BB: shinydashboard: Create Dashboards with “Shiny”. *R package version 071.* 2018 Reference Source

[22] Colaprico A Silva TC Olsen C: TCGAbiolinks: an R/Bioconductor package for integrative analysis of TCGA data. *Nucleic Acids Res.* 2016;44(8):e71. 10.1093/nar/gkv1507 26704973 PMC4856967

[23] Liu J Lichtenberg T Hoadley KA: An Integrated TCGA Pan-Cancer Clinical Data Resource to Drive High-Quality Survival Outcome Analytics. *Cell.* 2018;173(2):400–416.e11. 10.1016/j.cell.2018.02.052 29625055 PMC6066282

[24] Saltz J Gupta R Hou L: Spatial Organization and Molecular Correlation of Tumor-Infiltrating Lymphocytes Using Deep Learning on Pathology Images. *Cell Rep.* 2018;23(1):181–193.e7. 10.1016/j.celrep.2018.03.086 29617659 PMC5943714

[25] Tang J Shalabi A Hubbard-Lucey VM: Comprehensive analysis of the clinical immuno-oncology landscape. *Ann Oncol.* 2018;29(1):84–91. 10.1093/annonc/mdx755 29228097

[26] Tang J Hubbard-Lucey VM Pearce L: The global landscape of cancer cell therapy. *Nat Rev Drug Discov.* 2018;17(7):465–466. 10.1038/nrd.2018.74 29795477

[27] Tang J Pearce L O’Donnell-Tormey J: Trends in the global immuno-oncology landscape. *Nat Rev Drug Discov.* 2018;17(11):783–784. 10.1038/nrd.2018.167 30337722

[28] Yu JX Hubbard-Lucey VM Tang J: Immuno-oncology drug development goes global. *Nat Rev Drug Discov.* 2019;18(12):899–900. 10.1038/d41573-019-00167-9 31780841

[29] Chen T Guestrin C: XGBoost: A Scalable Tree Boosting System. In: *Proceedings of the 22Nd ACM SIGKDD International Conference on Knowledge Discovery and Data Mining.*KDD’16. New York, NY, USA: ACM.2016;785–794. 10.1145/2939672.2939785

[30] Gibbs DL: Robust classification of Immune Subtypes in Cancer. *bioRxiv.* 2020 10.1101/2020.01.17.910950

[31] Geman D d’Avignon C Naiman DQ: Classifying gene expression profiles from pairwise mRNA comparisons. *Stat Appl Genet Mol Biol.* 2004;3:Article19. 10.2202/1544-6115.1071 16646797 PMC1989150

[32] Jiang C Cao S Li N: PD-1 and PD-L1 correlated gene expression profiles and their association with clinical outcomes of breast cancer. *Cancer Cell Int.* 2019;19:233. 10.1186/s12935-019-0955-2 31516390 PMC6734479

[33] Padmanabhan R Kheraldine HS Meskin N: Crosstalk between HER2 and PD-1/PD-L1 in Breast Cancer: From Clinical Applications to Mathematical Models. *Cancers (Basel).* 2020;12(3):636. 10.3390/cancers12030636 32164163 PMC7139939

[34] Kurozumi S Inoue K Matsumoto H: Clinicopathological values of PD-L1 expression in HER2-positive breast cancer. *Sci Rep.* 2019;9(1):16662. 10.1038/s41598-019-52944-6 31723167 PMC6853939

