# Supplementary material for: CRI iAtlas: an interactive portal for immuno-oncology research
Source: F1000Res. 2020 Aug 24;9:1028. [Version 1] doi: 10.12688/f1000research.25141.1 (PMC7658727; doi:10.12688/f1000research.25141.1)
Supplement: Peer review history

# Peer review: Reviewer response for version 1

**DOI:** 10.5256/f1000research.27745.r73635

**Published:** 2020-12-04

**Authors:** Nathan E. Reticker-Flynn

**Affiliations:** 1 Department of Pathology, Stanford University School of Medicine, Stanford, CA, USA

In their manuscript, Eddy *et al*. describe the interactive data portal iAtlas that accompanies the authors’ 2018 publication in *Immunity*, “The Immune Landscape of Cancer”. In that seminal paper, the authors present a compendium of analyses they performed to interrogate the diversity of immune states across human cancers using publicly available data from The Cancer Genome Atlas (TCGA). While the *Immunity* manuscript described the identification and characterization of six distinct “Immune Subtypes” and highlighted examples of key distinguishing features of the data sets, the accompanying iAtlas tool enables users to explore these rich analyses on their own, using parameters of their choosing. Comprising over 11,000 patients across 33 cancer types, these data represent an exceptionally comprehensive patient cohort from which a wide range of immunological insights can be gleaned.

 iAtlas, which is available as both a web-based Shiny app and for offline implementation in R from GitHub, is a remarkably powerful and user-friendly portal through which researchers can easily interrogate the TCGA datasets for all of the immune analyses presented in their manuscript. The platform allows users to group their analyses by examining differences across TCGA cancer types, tumor subtypes (e.g. Luminal A vs. Her2 breast cancers), or their six defined Immune Subtypes. Furthermore, they have included the ability for users to define their own subsets and perform all of the included analyses across these subsets. The analyses within iAtlas include cellular readouts (e.g. degree of immune infiltration, fractions of particular leukocyte populations), molecular readouts (e.g. proliferation rates, gene expression, SNVs, CNAs, etc.), lymphocyte receptor (TCR/BCR) repertoires, network analyses, and tissue-level analyses (e.g. architectural characteristics). These variables can be investigated in the context of a wide range of parameters (e.g. clinical outcomes, correlations to driver gene mutations, etc.), designated as distinct modules, rendering the tool incredibly powerful with a near limitless number of potential questions to investigate in the field of tumor immunology. iAtlas is a remarkable tool with an extremely rich associated dataset whose elegant and straight-forward implementation render it accessible to researchers of all computational abilities.

 I have no major concerns with this manuscript. What follows are a list of comments, minor concerns, and suggestions that should not preclude acceptance of the manuscript but rather serve as potential items to include in future releases of iAtlas:

1. In the Cell Type Fractions of the Tumor Microenvironment module, it would be helpful to include an option for the user to select which CIBERSORT immune types to include as a custom “Cell Fraction Type”. For example, a user could select only memory resting CD4 T cells, memory activated CD4 T cells, naïve CD4 T cells, Tregs, and Tfh if they wanted to easily visualize the relative fractions of particular Th subsets within the CD4 compartment across groups. In some instances, it may be more biologically meaningful to compare these shifts within broad subsets without having to account for changes in unrelated populations (e.g. M2 macrophages).
2. In the Tumor Microenvironment module, when comparing leukocyte fraction to the total stromal fraction, there are typically a small number of samples where the leukocyte fraction exceeds the total stromal fraction, denoted as “estimation artifacts”. What is the source of these artifacts? It may help to include a description of this in the legend.
3. As a general comment, in a variety of instances it might be informative if there would be a way to include statistics between groups, though I recognize that doing so could be computationally intensive and invalid in many instances. Nonetheless, given the large sample sizes I am often left wondering if modest differences between groups are statistically meaningful and where inferences can be drawn (e.g. an increase in Treg fraction from 0.024 in the “Inflammatory” subtype to 0.029 in the “TGF-b Dominant” subtype).
4. The Data Description tab might be aided by including a sentence or phrase within the table stating what each variable is without requiring following all of the methods links. For example, I am assuming the BCR/TCR Shannon incorporates both the richness and evenness to depict the diversity of the repertoire, but it might be helpful to state the meaning of the terms in the table.
5. For the Clinical Outcomes, it might be useful to include “Immune Subtypes” as a variable when analyzing TCGA Study so that users can evaluate the survival probabilities depending on immune subtype for a given cancer.
6. There appears to be a minor issue wherein no results can be displayed for the Concordance Index of the Clinical Outcomes analyses for most, if not all, variables when using TCGA Study for the Sample Groups.
7. The TIL Maps module is quite impressive and the ability to examine the histology from each of the patients along with the calculated metrics is an outstanding feature. Nonetheless, when one clicks one of the violin plots, the annotation lists seems only to display an individual patient rather than the entire list of patients for that group.
8. The Immune association with driver gene analysis is a powerful tool whose insights have far-reaching implications and represent an active area of research in tumor immunology. While the interactive volcano plot is highly informative and useful for identifying genes of interest, it would also be useful to be able to run the analysis in the opposite direction. If possible, it would be informative to be able to select a driver gene and see where it lies in the various volcano plots and which of the variables are correlated with the mutation.

Are the conclusions about the tool and its performance adequately supported by the findings presented in the article?

Yes

Is the rationale for developing the new software tool clearly explained?

Yes

Is the description of the software tool technically sound?

Yes

Are sufficient details of the code, methods and analysis (if applicable) provided to allow replication of the software development and its use by others?

Yes

Is sufficient information provided to allow interpretation of the expected output datasets and any results generated using the tool?

Yes

Reviewer Expertise:

Tumor immunology, systems biology, immunotherapy, models of cancer, metastasis

I confirm that I have read this submission and believe that I have an appropriate level of expertise to confirm that it is of an acceptable scientific standard.

# Peer review: Reviewer response for version 1

**DOI:** 10.5256/f1000research.27745.r73636

**Published:** 2020-11-11

**Authors:** David L. Goode

**Affiliations:** 1 Computational Cancer Biology Program, Peter MacCallum Cancer Centre, Melbourne, Vic, Australia; 2 Sir Peter MacCallum Dept of Oncology, University of Melbourne, Melbourne, Vic, Australia

This article presents CRI iAtlas, a website with a suite of interactive tools for exploring and visualizing tumor immune microenvironment data collected by The Cancer Genome Atlas project. 

 It is based on robust, well-established R packages that are widely used for filtering, analysing and displaying large, multi-faceted data sets of the type used here.

 The design and implementation of the website are well described and the underlying code and data provided in readily accessible public repositories.

 CRI iAtlas will provide a valuable resource for the cancer immunology field. The tools it provides will enable a range of users to quickly and easily access and visualize complex genomics and immunology data sets. The interface is user friendly and well documented, particularly for those without bioinformatics training. 

 The detailed instructions certainly made it easier to understand what each tool did the first time I tried to use them. However, they also kind of clutter the interface. It would be nice to be able minimize those panels without minimizing the entire module.

 It would also be helpful to search the Driver Associations data for interactions involving certain genes of interest instead of having to scan through the volcano plots point by point.

 But those are minor quibbles. Overall this is a slick and powerful online tool that will be welcomed by the field.

Are the conclusions about the tool and its performance adequately supported by the findings presented in the article?

Yes

Is the rationale for developing the new software tool clearly explained?

Yes

Is the description of the software tool technically sound?

Yes

Are sufficient details of the code, methods and analysis (if applicable) provided to allow replication of the software development and its use by others?

Yes

Is sufficient information provided to allow interpretation of the expected output datasets and any results generated using the tool?

Yes

Reviewer Expertise:

Cancer Genomics, Bioinformatics

I confirm that I have read this submission and believe that I have an appropriate level of expertise to confirm that it is of an acceptable scientific standard.

# Peer review: Reviewer response for version 1

**DOI:** 10.5256/f1000research.27745.r70105

**Published:** 2020-10-05

**Authors:** Vincent Rouilly

**Affiliations:** 1 DATACTIX, Bordeaux, France

In this article, the authors introduce iAtlas, a web-based application that allows to browse a rich diversity of immune profiles, generated from the public TCGA dataset, and published in the landmark Immune Landscape study.

 Further than simply giving the possibility to replicate the published figures, the application provides a great flexibility to explore the entire PanImmune feature matrix through sophisticated and interactive tools. 

 The modular architecture and the features of the software are clearly explained. Detailed instructions are provided. And, all the necessary information is given to run the application.

 Its source code repository on github is well structured. The documentation is very comprehensive, as it gives ample information on the underlying methods, as well as how to extend the software application.

 It is a very valuable resource for the Immuno-oncology community.

Are the conclusions about the tool and its performance adequately supported by the findings presented in the article?

Yes

Is the rationale for developing the new software tool clearly explained?

Yes

Is the description of the software tool technically sound?

Yes

Are sufficient details of the code, methods and analysis (if applicable) provided to allow replication of the software development and its use by others?

Yes

Is sufficient information provided to allow interpretation of the expected output datasets and any results generated using the tool?

Yes

Reviewer Expertise:

Bioinformatics

I confirm that I have read this submission and believe that I have an appropriate level of expertise to confirm that it is of an acceptable scientific standard.
